# Resourcefulness training on cancer-related fatigue and fear of cancer recurrence in patients undergoing colorectal cancer chemotherapy: a quasi-experimental study

**DOI:** 10.3389/fpubh.2025.1733348

**Published:** 2026-01-12

**Authors:** Liyan Gao, Yaping Zheng, Nafei Han, Xiaoyan Wu, Huadi Yuan

**Affiliations:** Department of Nursing, The Second Affiliated Hospital, Zhejiang University School of Medicine, Hangzhou, China

**Keywords:** cancer-related fatigue, colorectal cancer, fear of cancer recurrence, resourcefulness theory, resourcefulness training

## Abstract

**Background:**

Patients with colorectal cancer commonly experience problems, such as fear of cancer recurrence and cancer-related fatigue, which seriously affect their physical and mental health. This study aimed to investigate the applicability of resourcefulness training on patients with colorectal cancer undergoing chemotherapy and explore its impact on patients’ cancer-related fatigue scores, fear of cancer recurrence scores, and resourcefulness levels.

**Materials and methods:**

Sixty chemotherapy patients who attended a tertiary comprehensive hospital in Zhejiang Province between May and July 2025 were enrolled in the study and divided into two groups according to the ward. The control group received routine care, while the intervention group received a four-week resourcefulness intervention, in addition to routine care. Pre- and post-intervention differences between the two groups were compared using the Cancer Fatigue Scale, the Fear of Recurrence Questionnaire, and the Resourcefulness Scale. Descriptive analysis was used, and data were analyzed using the Student’s t-test, Fisher’s exact test, Mann–Whitney U test, paired t-test, or Wilcoxon signed-rank test, depending on data normality. Analysis of covariance was used to compare the two groups after the intervention.

**Results:**

The total scores for fear of cancer recurrence and cancer-related fatigue in both groups decreased compared to those before the intervention (*p* < 0.001), while the total score for resourcefulness increased compared to those before the intervention (*p* < 0.001). Inter-group comparisons revealed that the fear of cancer recurrence score was significantly lower (*F* = 140.734, *p* < 0.001, *η*^2^ = 0.712), the resourcefulness score was higher (*F* = 164.940, *p* < 0.001, *η*^2^ = 0.743) and the fatigue score was lower (*F* = 205.589, *p* < 0.001, *η*^2^ = 0.783) in the intervention group than in the control group. However, no statistically significant difference was observed in the cognitive fatigue dimension between the two groups (*p* = 0.132).

**Conclusion:**

Compared with conventional care, resourcefulness training can reduce physical and emotional fatigue levels in patients undergoing chemotherapy for colorectal cancer, decrease the fear of disease progression, and improve patients’ resourcefulness. It is suitable for colorectal cancer patients undergoing chemotherapy. However, the effectiveness in improving cognitive fatigue remains unclear.

## 1 Introduction

The 2022 global cancer burden data from the International Agency for Research on Cancer revealed that colorectal cancer (CRC) accounted for 1,926,118 new cases and 903,859 deaths worldwide. CRC ranks third in incidence and second in mortality rates among all malignant tumors (1). Every year, 376,000 new CRC cases are diagnosed in China, with 191,000 deaths (2). Surveys have indicated that China has the highest demand for chemotherapy among patients with CRC (3). After chemotherapy, cancer-related fatigue (CRF) is a common symptom in patients with CRC (4). According to the concept proposed by the National Comprehensive Cancer Network, CRF is a subjective feeling of fatigue or tiredness related to cancer or cancer treatment that is disproportionate to recent activities and often disrupts daily function. It is the sixth vital sign of cancer (5). Chemotherapy is a major risk factor for CRF in patients with CRC (6). CRF reaches its peak immediately in patients with CRC receiving adjuvant chemotherapy and remains common in patients receiving chemotherapy for 2 years (7). A longitudinal study of patients with CRC found that physical fatigue and total fatigue scores increased during chemotherapy, while emotional and cognitive fatigue scores increased significantly 3 months after chemotherapy and then remained stable (8). The chemotherapy stage is a high-risk time point for patients with CRC to develop CRF, which urgently requires attention from medical staff.

CRF is a multidimensional phenomenon encompassing physical, cognitive, and emotional fatigue (9). According to the fear of cancer recurrence (FCR) model proposed by Lee-Jones, symptoms, as the patient’s intuitive somatic manifestation, become an important internal trigger for FCR (10). Patients undergoing chemotherapy are prone to interpret changes in physical, emotional, and cognitive symptoms as signs of cancer recurrence or progression, falling into worries about the follow-up of the disease, triggering FCR. FCR is an individual’s fear and concern about the possibility of cancer recurrence, metastasis, or progression (11). It is a common response to cancer diagnosis and related treatments and can exist stably throughout treatment and survival trajectories (12). Some researchers have studied the relationship between CRF and FCR in patients with cancer. Esser et al. (13) found that FCR mediates the relationship between CRF and quality of life in patients with malignant blood diseases, suggesting that future researchers can help buffer the adverse effects of CRF by addressing FCR in patients. Trudel et al. (14) revealed that among young cancer patients aged 55 years or below, the presence of CRF exacerbates FCR, and the relationship between the two may strengthen over time. Patients with CRC generally experience problems such as FCR and CRF, which seriously affect their physical and mental health. However, there is still a lack of research on this group, and patients with CRC require immediate interventions to improve their physical and mental health.

With the development of positive psychology, resourcefulness has emerged as an important positive psychological resource that helps individuals cope positively (15). Meichenbaum first proposed the concept of learned resourcefulness (16), resourcefulness is the cognitive skill of self-control used by individuals to regulate their responses to stressful life events. Self-control is the core of learned resourcefulness and is divided into remedial, ameliorative, and experiential self-control. Learned resourcefulness emphasizes the use of internal resources by individuals. Therefore, Rapp et al. (17) proposed social resourcefulness in seeking help from others. Currently, most researchers agree with the concept of resourcefulness proposed by Zauszniewski (18). Resourcefulness includes two abilities: the ability of individuals to complete tasks independently in daily life, known as personal resourcefulness, and the comprehensive ability of individuals to seek help from the outside when they cannot complete tasks independently, known as social resourcefulness. Personal resourcefulness is further divided into three components: redressive self-control (individuals use proactive self-direction to control existing or impending problems to restore normal functioning), modified problem-solving skills (individuals alter old, habitual thinking, change problem-solving strategies, and delay the need for immediate gratification), and perceived self-efficacy (a reflection of an individual’s belief in effective coping and a self-assessment of their ability to achieve desired goals). Social resourcefulness includes formal assistance from medical professionals and informal assistance from family and friends.

Resourcefulness reflects an individual’s ability to cope with stress and use resources (19). Resourcefulness training has proven to be an effective cognitive-behavioral intervention method (20). Therefore, this study used resourcefulness as a theoretical guide and applied resourcefulness training to patients with CRC during chemotherapy to reduce the adverse effects of CRF and FCR, provide ideas and references for developing mental health interventions for cancer patients during chemotherapy, and enhance the adaptability and scientific validity of intervention measures based on resourcefulness theory in cancer populations.

## 2 Materials and methods

### 2.1 Design

**Figure 1:**
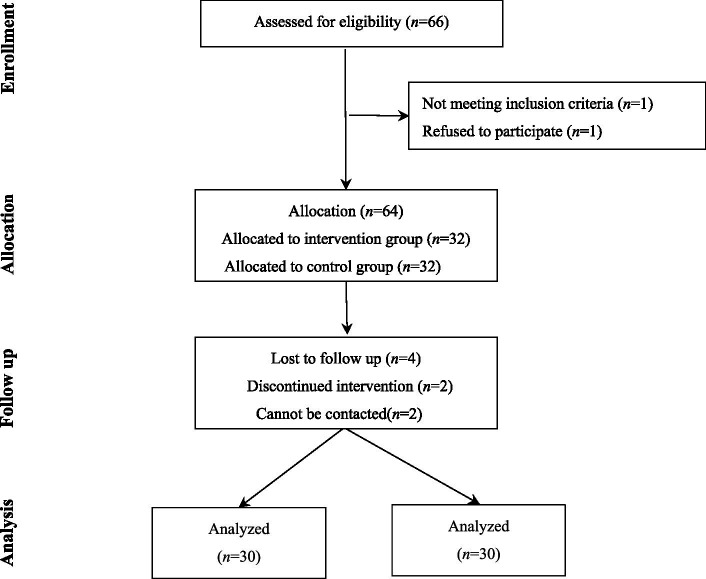
Flow diagram.

Convenience sampling was used to select patients who underwent chemotherapy in our oncology department between May and July 2025. The sample size was calculated using formula n_1_ = n_2_ = 2 [(t_α/2_ + t_β_) *σ*/*δ*] ^2^, where t_α/2_ = 1.96 and t_β_ = 1.28 (21), σ: the standard deviation of two populations, δ: the mean of two populations. The Fear of Progression Questionnaire-Short Form (FoP-Q-SF) was used as the primary outcome measure. Based on a preliminary pilot study, *σ* = 4.44, *δ* = 4.00, and σ/δ = 1.11 were calculated, indicating the requirement of 26 patients per group. Allowing a 10% dropout rate, the intervention and control groups each comprised 29 patients at least. The intervention was divided into two groups: wards 1 and 2, with a total of 66 patients. Patients were grouped based on their ward of admission, and were assigned to the intervention (32 patients in Ward 1) or control (32 patients in Ward 2) groups. Both wards implemented homogeneous management of patient treatment and nursing. However, 4 participants were excluded from the statistical analysis because the discontinued intervention and cannot be contacted (Figure 1). To avoid data contamination, patients were asked by a nursing master student(not involved in the patient intervention and was unaware of the study group) whether they had been exposed to resourcefulness training after completing the intervention. Those who reported exposure were excluded; however, no contamination was detected in the survey.

The inclusion criteria were as follows: (1) Diagnosis of CRC by postoperative histopathological examination; (2) age ≥ 18 years; (3) undergoing chemotherapy with a 14-day cycle; (4) medical condition permitting, personal access to a smartphone, and the ability to use WeChat or the ability to use it after guidance; (5) voluntary participation with informed consent. The exclusion criteria included the following: (1) presence of severe cognitive, psychiatric, or language communication disorders; (2) severe organic heart, brain, or lung lesions or severe complications that could affect their responses; (3) unawareness of their underlying medical condition; (4) participation in other psychological treatments. This study was approved by the Ethical committee of the Second Affiliated Hospital of Zhejiang University School of Medicine (Approval no. I20240278) and complied with the Declaration of Helsinki (22). All information provided by the participants remained strictly confidential.

### 2.2 Nursing interventions

#### 2.2.1 Control group

The control group received routine care, which included an introduction to the ward environment, distribution of a routine oncology education manual, guidance about medication knowledge, healthy diet, and disease-related health education for patients, prevention, identification, and response to common adverse reactions to chemotherapy, assessment and counseling of common psychological problems during chemotherapy, explanation of discharge precautions, and follow-up of catheterization and medication after discharge.

#### 2.2.2 Experimental group

A intervention team consisting of six members, all proficient in resourcefulness training: one head nurse, two charge nurses, one registered psychologist, and two nursing master’s students. The specific division of labor was as follows: the head nurse oversaw the guidance and overall quality of the strategic intervention plan; the registered psychologist supervised the intervention implementation process; the two charge nurses assisted in the selection of research subjects, answering questions regarding disease treatment and nursing care; and the nursing master’s student was responsible for implementing the strategic intervention and collecting patient data. Data collection was handled by a nursing master’s student who was not involved in the patient strategic intervention and was unfamiliar with the research subject groups.

The majority of published intervention trials for CRF involve in-person interventions delivered by trained providers, which restricts access and limits the reach of many interventions (23). Considering patient compliance, the intervention lasted for 4 weeks with weekly sessions, which was consistent with existing research (24, 25). Face-to-face individual counseling sessions were limited to 20–30 min each, and WeChat online group activities were limited to 30–45 min each. WeChat notifications were also sent at least once a week. Additionally, online individualized intervention sessions were conducted via WeChat using audio, video, or phone calls, each lasting 5–10 min. Besides routine care, the intervention group received resourcefulness training. An intervention manual was distributed. The structure was designed based on previous studies (26). The overall framework of the program is presented in Table 1, and its specific content is presented in Table 2.

**Table 1:** Overall framework of the resourcefulness training for patients with CRC during chemotherapy.

Main framework	Secondary framework	Conceptual Implications	Overall goal
Personal resourcefulness	Redressive self-control	Through positive self-guidance, to confront current or potential problems and proactively manage emotions	To master the skills of emotional control and understand the related knowledge of CRC in chemotherapy
	Modified problem-solving skills	To break through inherent thought and behavioral patterns, optimize problem-solving strategies, interrupt old thinking habits, and cultivate the ability to delay direct gratification	To assist patients in adapting to the discomfort and complications of chemotherapy
	Perceived self-efficacy	To strengthen an individual’s coping beliefs through verbal persuasion, direct experience, and vicarious experience	To help patients maintain a positive attitude, and build confidence in their fight against the disease
Social resourcefulness	Informal assistance	Receive support and assistance from family and friends	To master and apply family and interpersonal relationship skills flexibly
	Formal assistance	Receive help from hospitals, medical personnel, and other professional institutions or individuals	To help patients understand and acquire professional social support

**Table 2:** Resourcefulness training program for patients with CRC during chemotherapy.

Time	Theoretical framework	Intervention goals	Intervention content	Intervention form
Week 1	Personal resourcefulness	① Patients were familiar with their own emotions and mastered the skills of emotional control ② Guide patients to face the disease and improve their disease awareness	① Assess the patient’s emotional state, teach and guide the breathing relaxation training ② Push CRC videos and pictures (cause, pathogenesis,clinical manifestations,chemotherapy regimens,common adverse reactions)	Oral explanation Push on WeChat
	Social resourcefulness	Introducing informal and formal assistance	① Guide patients to express their emotions to their families, enhance family support ② Forwarding official and authoritative links within the group after screening	Discuss Push on WeChat
Week 2	Personal resourcefulness	① Learn to manage chemotherapy-related reactions ② Help patients change and optimize problem-solving strategies	① Push videos and pictures concerning the treatment, prevention of chemotherapy-related adverse reactions, as well as home management during chemotherapy, to help patients define and solve problems. ② Follow up and list the disease problems, identify the primary problems, and provide targeted guidance	Push on WeChat Follow-up
	Social resourcefulness	Learn to use formal assistance	① Answer questions by Professional medical staff. Those with severe symptoms were advised to seek medical treatment. ② Follow up and dynamically assess their use of formal assistance	Oral explanation Follow-up
Week 3	Personal resourcefulness	① Strengthen patients’ confidence and self-efficacy in treatment ② Improve the source of patients’ anti-cancer beliefs	① Invite optimistic patients to share their anti-cancer experiences. For patients who were not active, communicate individually ② Pushes patients with CRC anti-cancer stories to read	Discuss Push on WeChat
	Social resourcefulness	Learn to use informal assistance	① Understand the patient’s current interpersonal and family relationships, and social resources ② Explain interpersonal communication, family relationship skills, and common medical and social resources	Discuss Explain
Week 4	Personal resourcefulness	Strengthen personal resourcefulness skills	① Assess the patient’s emotional state, understand the strength of the patient’s anti-cancer beliefs, summarize and provide guidance to the patient’s questions ② Summarize the content of the past three weeks and promote the comprehensive skills application	Follow-up Discuss
	Social resourcefulness	Strengthen formal and informal assistance skills	① Assess the patient’s use of formal and informal assistance and provide further guidance if necessary ② Summarize formal and informal assistance strategies to consolidate	Follow-up Push on WeChat

To ensure patient adherence, we manage it from the following 3 aspects: ① face-to-face individual counseling: introducing ourselves to patients, providing a detailed introduction to the activity, and obtaining informed consent; maintaining proactive contact with patients, fully respecting their personal wishes, exchanging WeChat and phone numbers, inviting them to follow our WeChat platform and join the online group chat, and uploading course materials to the WeChat platform. ② WeChat online group activities: researchers informed patients of the activity time in advance, reminded them again in the group on the day of the activity, and promptly answered their questions. ③ WeChat push notifications: user analytics were used to understand patients’ usage frequency and cumulative message views, encouraging patients to participate in the entire 4-week activity.

After each intervention, the team members conducted a debriefing, reviewed the problems encountered during the implementation of the resourcefulness intervention, and proposed further modifications. For example, during the first face-to-face individual counseling, patients showed little interest in theoretical knowledge, so stories and cases were incorporated into the introduction. The research team jointly discussed the course content to ensure it was easy to understand and maximize the course’s effectiveness. During online WeChat push notifications, patients preferred audiovisual methods, so the push content combined text, images, videos, and audio to ensure the fidelity of the research.

### 2.3 Instruments

#### 2.3.1 General characteristics questionnaire

The questionnaire was developed by the researchers following a group discussion and included questions about gender, age, educational level, cancer type, disease stage, and stoma status.

#### 2.3.2 FoP-Q-SF

FoP-Q-SF was compiled by German scholars Mehnert et al. (11) and translated into Chinese by Wu et al. (27). It was divided into two dimensions: physical health (6 items) and social family (6 items). The scores range from “never” to “always” and are scored on a scale of 1–5 points. A total score of 34 points or more is considered indicative of a disorder affecting the body’s psychological function (28). The Cronbach’s *α* for the Chinese version of the FoP-Q-SF was 0.883 (27).

#### 2.3.3 Resourcefulness scale

RS was compiled by Zauszniewski et al. (18) in 2006 and translated into Chinese by Ke et al. (29) in 2015. It is divided into two dimensions: personal resourcefulness (16 items) and social resourcefulness (12 items). Each item is rated on a 6-point Likert scale, ranging from 0 (extremely non-descriptive of one’s behavior) to 5 (completely descriptive). The higher the score, the higher the resourcefulness of the individual (30). The RS is widely applied in the Chinese context (31) and has good reliability and validity. The Cronbach’s *α* for the Chinese version of the RS was 0.91 (29).

#### 2.3.4 Cancer fatigue scale

CFS was compiled by Okuyama and colleagues (32) to evaluate the degree of fatigue in patients with cancer. It was translated into Chinese by Zhang et al. (33). It comprises 15 items, categorized into three dimensions: physical (7 items), affective (4 items), and cognitive (4 items). It uses a 5-point Likert rating method: 1 point = not at all; 2 points = a little; 3 points = some; 4 points = quite; 5 points = very. The scores for physical, emotional, and cognitive fatigue were in the range of 0–28, 0–16, and 0–16, respectively. The physical fatigue dimension score = the sum of the scores of the items in the physical dimension −7; The affective fatigue dimension score = 20—the sum of the scores of the items in the affective dimension; The cognitive fatigue dimension score = the sum of the scores of the items in the cognitive dimension—4; The total CFS score is the sum of the scores of the three dimensions, ranging from 0 to 60 points. A higher score indicates more severe CRF (34). The internal consistency of the CFS dimensions with the total scale was measured by Cronbach’s *α* ranging from 0.63 to 0.86, and the reliability ranged from 0.55 to 0.77 (33).

### 2.4 Statistical analysis

The Statistical Package for the Social Sciences software (version 25.0; IBM Corp., Armonk, NY, United States) was used to analyze questionnaire data. Continuous data are expressed as mean ± standard deviation or median with interquartile range, while categorical data are expressed as rates and component ratios. Baseline data between the two groups were compared using independent sample *t*-tests, chi-squared tests, Fisher’s exact test. Within-group comparisons were performed using paired *t-*tests or Wilcoxon signed-rank tests, depending on the normality of the data. Between-group comparisons were performed using independent sample *t-*tests or Mann–Whitney U tests. Analysis of covariance was used to compare the two groups after the intervention, with pre-intervention scores as covariates, post-intervention scores as dependent variables, and group as a fixed factor. *p* < 0.05 was considered statistically significant.

## 3 Results

### 3.1 Characteristics of the participants

The demographic characteristics and clinical data of the two patient groups were analyzed. The two patient groups were balanced and comparable in terms of baseline data (*p* > 0.05; Table 3).

**Table 3:** Comparison of general information between the two groups of patients with CRC.

Item	Classification	Intervention group (*n* = 30)	Control group (*n* = 30)	*t/x^2^/*Fisher’s exact test	*p*-value
Age (years)	—	52.40 ± 9.71	54.90 ± 8.36	−1.069	0.290
Gender	Male	18 (60.0)	18 (60.0)	—	1.000
	Female	12 (40.0)	12 (40.0)		
Residence	Rural	10 (33.3)	12 (40.0)	0.288	0.866
	Town	9 (30.0)	8 (26.7)		
	City	11 (36.7)	10 (33.3)		
BMI	Thin	1 (3.3)	2 (6.7)	0.619	0.892
	Normal	17 (56.7)	18 (60.0)		
	Overweight	9 (30.0)	7 (23.3)		
	Obesity	3 (10.0)	3 (10.0)		
Marriage	Married	29 (96.7)	29 (96.7)	—	1.000
	Unmarried	1 (3.3)	1 (3.3)		
Professional status	Unemployed	2 (6.7)	2 (6.7)	0.382	0.826
	Employed	8 (26.7)	6 (20.0)		
	Retired	20 (66.7)	22 (73.3)		
Education level	Primary school and below	7 (23.3)	8 (26.7)	0.622	0.733
	Junior high school	8 (26.7)	10 (33.3)		
	High school and above	15 (50.0)	12 (40.0)		
Cancer type	Colon cancer	19 (63.3)	18 (60.0)	0.071	0.791
	Rectal cancer	11 (36.7)	12 (40.0)		
Disease stage	Stage III	3 (10.0)	2 (6.7)	0.000	1.000
	Stage IV	27 (90.0)	28 (93.3)		
Stoma	Have	3 (10.0)	2(6.7)	—	1.000
	None	27 (90.0)	28 (93.3)		
Disease course	<6 months	6 (20.0)	9 (30.0)	1.691	0.429
	6-12 months	5 (16.7)	7 (23.3)		
	>12 months	19 (63.3)	14 (46.7)		
Hypertension	Have	9 (30.0)	10 (33.3)	0.077	0.781
	None	21 (70.0)	20 (66.7)		
Diabetes	Have	3 (10.0)	4 (14.3)	—	1.000
	None	27 (90.0)	26 (86.7)		

### 3.2 Effect of resourcefulness training on CRF in patients with CRC

Pre-intervention, there were no statistically significant differences in any of the CFS scale indicators between the two groups (*p* > 0.05). Post-intervention, the total score and each dimension of the fatigue scale were significantly lower in the intervention group than in the pre-intervention (*p* < 0.001). Analysis of covariance showed that after the intervention, except for the cognitive fatigue dimension which had no statistical significance (*p* = 0.132), the total score of the CRF scale in the intervention group was significantly lower than that in the control group (*F* = 205.589, *p* < 0.001, *η*^2^ = 0.783). The improvement in all other dimensions was better than that in the control group, and the differences were statistically significant (*p* < 0.001; Table 4).

**Table 4:** Comparison of CRF levels between the two groups of patients with CRC.

Classification	Time	Intervention group (*n* = 30) ^−^*x ± s/M*50 (*M* 25, *M* 75)	Control group (*n* = 30) ^−^*x ± s*/*M*50 *(M* 25, *M* 75)	*t/F* value	*p*-value
Physical fatigue	Pre-intervention	13.07 ± 1.99	13.80 ± 2.35	−1.300	0.199
	Post-intervention	4.97 ± 2.08	11.13 ± 2.40	143.496	<0.001
	*t*-value	21.225	7.688	—	—
	*p*-value	< 0.001	< 0.001	—	—
Affective fatigue	Pre-intervention	9.40 ± 1.30	9.50 ± 1.70	−0.256	0.799
	Post-intervention	4.83 ± 1.34	7.50 ± 1.80	80.726	<0.001
	*t*-value	18.794	11.148	—	—
	*p*-value	< 0.001	< 0.001	—	—
Cognitive fatigue	Pre-intervention	0 (0, 1)	0 (0, 1)	−1.199	0.230
	Post-intervention	0 (0, 0)	0 (0, 0)	2.331	0.132
	*Z* value	−3.127	−2.333	—	—
	*p*-value	0.002	0.020	—	—
CRF total score	Pre-intervention	23.07 ± 2.69	23.80 ± 3.76	−0.868	0.389
	Post-intervention	9.97 ± 2.98	18.90 ± 3.74	205.589	<0.001
	*t*-value	31.269	11.262	—	—
	*p*-value	< 0.001	< 0.001	—	—

### 3.3 Effect of resourcefulness training on the FCR in patients with CRC

Pre-intervention, there was no statistically significant difference in the total score or each dimension of FCR between the two patient groups with CRC (*p* > 0.05). Post-intervention, the total score and each dimension of FCR decreased in both groups (*p* < 0.05). Analysis of covariance showed that the total score of FCR in the intervention group was significantly lower than that in the control group after intervention (*F* = 140.734, *p* < 0.001, *η*^2^ = 0.712), and the improvement in two dimensions was significantly better in the intervention group than in the control group (*p* < 0.001; Table 5).

**Table 5:** Comparison of FCR between the two groups of patients with CRC.

Classification	Time	Intervention group (*n* = 30) ^−^*x ± s*	Control group (*n* = 30) ^−^*x ± s*	*t/F*-value	*p*-value
Physical health	Pre-intervention	22.93 ± 2.07	22.83 ± 2.10	0.186	0.853
	Post-intervention	16.90 ± 2.04	21.00 ± 2.21	115.650	<0.001
	*t*-value	22.079	5.966	—	—
	*p*-value	< 0.001	< 0.001	—	—
Social family	Pre-intervention	15.87 ± 2.51	15.10 ± 2.19	1.260	0.213
	Post-intervention	12.47 ± 1.55	13.60 ± 2.40	18.400	<0.001
	*t*-value	9.872	5.642	—	—
	*p*-value	< 0.001	< 0.001	—	—
FCR total scores	Pre-intervention	38.80 ± 3.30	37.83 ± 2.99	1.189	0.239
	Post-intervention	29.40 ± 2.54	34.57 ± 3.30	140.734	<0.001
	*t*-value	25.395	8.521		
	*p*-value	< 0.001	< 0.001	—	—

### 3.4 Effect of resourcefulness training on resourcefulness in patients with CRC

Pre-intervention, there were no statistically significant differences in the RS scale between the two patient groups with CRC (*p* > 0.05). Analysis of covariance showed that the total RS score of the intervention group was significantly higher than that of the control group after intervention (*F* = 164.940, *p* < 0.001, *η*^2^ = 0.743), and the improvement in two dimensions was better than that of the control group (*p* < 0.001; Table 6).

**Table 6:** Comparison of resourcefulness between the two groups of patients with CRC.

Classification	Time	Intervention group (*n* = 30) ^−^*x ± s*	Control group (*n* = 30) ^−^*x ± s*	*t/F*-value	*p*-value
Personal resourcefulness	Pre-intervention	38.00 *±* 2.70	37.37 *±* 3.36	0.805	0.424
	Post-intervention	47.13 *±* 3.80	41.83 *±* 3.61	78.098	< 0.001
	*t*-value	−21.070	−13.929	—	—
	*p*-value	< 0.001	< 0.001	—	—
Social resourcefulness	Pre-intervention	41.53 *±* 2.57	40.23 ± 3.50	1.640	0.106
	Post-intervention	52.83 ± 3.12	45.43 ± 3.22	95.068	< 0.001
	*t*-value	−18.451	−13.621	—	—
	*p*-value	< 0.001	< 0.001	—	—
RS total scores	Pre-intervention	79.53 ± 2.49	77.57 ± 5.89	1.688	0.099
	Post-intervention	99.93 ± 3.95	87.27 ± 5.91	164.940	< 0.001
	*t*-value	−34.903	−23.033	—	—
	*p*-value	< 0.001	< 0.001	—	—

## 4 Discussion

Resourcefulness is a positive psychological and behavioral factor that enables patients to cope with disease and maintain their health status (35). It can buffer the impact of the physical and mental discomfort caused by the disease on patients. Patients with high resourcefulness often experience lower chemotherapy-related fatigue (36). The results of the intra-group comparison in this study revealed that the total CRF score and each dimension of the two groups decreased. The inter-group comparison revealed that, except for the cognitive fatigue dimension, the intervention group displayed a greater reduction in CRF than the control group, with statistically significant differences.

According to the report, CRF was prevalent in all 15 investigated cancer entities even 2 years after diagnosis (37). Physical fatigue is the subjective perception of physical performance, which objectively manifests as a need for multiple breaks or increased sleep during activities. Resourcefulness training provides knowledge about CRC and chemotherapy, as well as the treatment of adverse reactions and home management. It enhances the way patients obtain treatment information during chemotherapy, assists patients in solving problems, and reduces the adverse effects of the disease on their bodies. Simultaneously, resourcefulness, as an individual’s ability to cope with adversity, can buffer the negative impact of stress on mental health (38), help patients build confidence, increase self-efficacy to face treatment positively, enhance family support, and reduce affective fatigue. Cognitive fatigue is the subjective experience of sleepiness and tiredness at the cognitive level in patients with cancer. A previous prospective study (39) indicated that increased CFS levels were strongly correlated with a range of cognitive-behavioral factors. Individuals with poor cognitive abilities were more prone to extreme thinking, which can easily lead to fatigue. Because our resourcefulness training programs primarily focus on emotional control, problem-solving, and social support, interventions regarding focus, memory, and cognitive processing may not be in-depth and comprehensive enough,which means that psychological interventions were not likely to change the cognitive fatigue easily. Future improvements are needed to incorporate cognitive fatigue into intervention designs, thereby further enhancing the effectiveness of resourcefulness training.

Data shows that even with curative surgery, up to 50% of patients with CRC may experience metastasis or recurrence (40). A cross-sectional survey of 10,969 patients with CRC revealed that approximately 50% of patients with CRC were worried about their disease would develop FCR (41). This suggests that FCR is common in patients with CRC, and negative psychological problems are more prominent (42). In this study, patients had a relatively prolonged disease course, and the negative emotion of FCR still requires attention.

Within-group comparisons showed that both groups experienced a decrease in total FCR score and all dimensions. Between-group comparisons showed that the intervention group exhibited a greater degree of reduction in total FCR score and all dimensions compared to the control group. The reasons for the reduction in FCR levels in patients with CRC were investigated. Illness perception, social constraints, and maladaptive cognitive emotion regulation strategies were found to be the leading contributors to FCR in CRC patients (43). Resourcefulness is an adjustable intermediate variable between cognition and behavior, which can effectively cope with the impact of internal and external stress events and manage negative thoughts through positive thinking (44). The resourcefulness training permeates knowledge related to the disease and chemotherapy, helps patients identify the problems that bother them, teaches them emotional management skills, breathing and relaxation training skills, and problem-solving methods, improves their ability to manage their own disease during chemotherapy, and enables them to believe that their disease progression and the occurrence of chemotherapy complications can be controlled, thereby alleviating uncertainty about the disease (15). Resourcefulness training informed patients of formal social assistance resource acquisition skills, interpersonal communication, family conflict resolution skills, and encouraged patients to self-disclose to each other, and interact with medical staff in the group. It invited optimistic patients to share their experiences with fellow patients battling cancer. Patients can establish a sense of self-efficacy, and effectively use their subjective initiative to cope with the fear of disease progression (45). Our study validates the efficacy of resourcefulness training in improving FCR in CRC patients. Based on the results of this study, we can develop and promote standardized psychological intervention for CRC chemotherapy patients guided by the theory, and expand the coverage of intervention measures by remote nursing and other means, so that more patients can receive timely and effective psychological support.

Intra-group comparisons showed improvements in total RS scores and all dimensions in both groups. Inter-group comparisons showed that the intervention group exhibited greater improvements in RS scores and all dimensions than the control group. Consistent with the results of the resourcefulness training study conducted by Huang et al. (46) on patients with nasopharyngeal carcinoma. In the routine nursing of the control group, most content focused on general health education and basic nursing care during chemotherapy, which nurses typically provided without fully considering the actual needs of patients, and lacked targeted improvement strategies.

The intervention group attempted to combine the framework concept of resourcefulness with routine chemotherapy education and apply it to clinical practice. The intervention content was formulated around the core elements of resourcefulness (47). The resourcefulness training program can actively guide patients with CRC in developing internal and external resources during chemotherapy, help them restructure their emotions, improve their problem-solving abilities, and establish a sense of self-efficacy. It can teach patients interpersonal communication and family help-seeking skills, guide patients to maximize the use of social channels and professional means to seek medical help, give full play to the linkage between patients, their families, primary caregivers, and medical staff, promote the improvement of personal and social resourcefulness and assist patients with CRC to make a smooth transition during chemotherapy. In this study, the resourcefulness training intervention not only aligned with the patients’ needs but also closely matched the intended goals. The implementation of resourcefulness theory contributed to making the intervention as methodical and all-encompassing as feasible, while also substantiating its efficacy.

## 5 Conclusion

This study demonstrated that resourcefulness training can significantly improve the resourcefulness of patients with CRC undergoing chemotherapy and reduce FCR and CRF. Resourcefulness interventions are an important cognitive-behavioral approach. The application of resourcefulness theory has broadened the clinical psychological nursing intervention model for patients undergoing chemotherapy for CRC and provides a new research perspective. As patients have a short hospital stay during chemotherapy and need to train at home, group training conducted through WeChat may impact the effectiveness of the intervention. Because our study used convenience sampling, there may be selection bias; all the scales involved were subjective, and objective indicators need to be included. The intervention period was short and the follow-up time was not long, so the long-term effects of the resourcefulness training could not be fully observed. All patients were from the same hospital, the sample size was small, and the representativeness of the sample may be insufficient, which, to a certain extent, limits the universality of the research results. In the future, it will be necessary to improve the intervention plan, establish a standard intervention process, and conduct multi-center, large-scale studies.

## Data Availability

The raw data supporting the conclusions of this article will be made available by the authors, without undue reservation.
